# Connective Tissue Growth Factor (CTGF) Expression Modulates Response to High Glucose

**DOI:** 10.1371/journal.pone.0070441

**Published:** 2013-08-12

**Authors:** Leighton R. James, Catherine Le, Heather Doherty, Hyung-Suk Kim, Nobuyo Maeda

**Affiliations:** 1 Department of Medicine, University of Florida, Jacksonville, Florida, United States of America; 2 Department of Medicine, University of Texas Southwestern Medical Center, Dallas, Texas, United States of America; 3 Department of Pathology and Laboratory Medicine, University of North Carolina at Chapel Hill, Chapel Hill, North Carolina, United States of America; INSERM, France

## Abstract

Connective tissue growth factor (CTGF) is an important mediator of fibrosis; emerging evidence link changes in plasma and urinary CTGF levels to diabetic kidney disease. To further ascertain the role of CTGF in responses to high glucose, we assessed the consequence of 4 months of streptozotocin-induced diabetes in wild type (+/+) and CTGF heterozygous (+/−) mice. Subsequently, we studied the influence of glucose on gene expression and protein in mice embryonic fibroblasts (MEF) cells derived from wildtype and heterozygous mice. At study initiation, plasma glucose, creatinine, triglyceride and cholesterol levels were similar between non-diabetic CTGF+/+ and CTGF+/− mice. In the diabetic state, plasma glucose levels were increased in CTGF+/+ and CTGF+/− mice (28.2 3.3 mmol/L vs 27.0 3.1 mmol/L), plasma triglyceride levels were lower in CTGF+/− mice than in CTGF+/+ (0.7 0.2 mmol/L vs 0.5 0.1 mmol/L, p<0.05), but cholesterol was essentially unchanged in both groups. Plasma creatinine was higher in diabetic CTGF+/+ group (11.7±1.2 vs 7.9±0.6 µmol/L p<0.01), while urinary albumin excretion and mesangial expansion were reduced in diabetic CTGF+/− animals. Cortices from diabetic mice (both CTGF +/+ and CTGF +/−) manifested higher expression of CTGF and thrombospondin 1 (TSP1). Expression of nephrin was reduced in CTGF +/+ animals; this reduction was attenuated in CTGF+/− group. In cultured MEF from CTGF+/+ mice, glucose (25 mM) increased expression of pro-collagens 1, IV and XVIII as well as fibronectin and thrombospondin 1 (TSP1). In contrast, activation of these genes by high glucose was attenuated in CTGF+/− MEF. We conclude that induction of *Ctgf* mediates expression of extracellular matrix proteins in diabetic kidney. Thus, genetic variability in CTGF expression directly modulates the severity of diabetic nephropathy.

## Introduction

CTGF (CCN2) is a pleiotropic growth factor belonging to the 30–40 kDa CCN family of proteins which includes Cyr61, NOV and WISP1. This family of proteins exhibits a diverse array of cellular effects (mitogenesis, apoptosis, regulation of extracellular matrix (ECM) physiology, osteogenesis, embryogenesis, angiogenesis and tumorigenesis), are critical for growth, development and differentiation and are responsive to various intra- and extracellular stimuli (please see references [1]–[4] for detailed review). CTGF, product of the gene C*tgf*, is considered as an important mediator of fibrosis [4], [5], yet many of the signals involved in this process remain unknown.

High glucose induces *Ctgf* expression in human vascular smooth muscle cells (VSMC) and this is accompanied by increased expression of fibronectin and collagen type 1 [6]. In non-primate human models with diabetes, glomerular and tubular *ctgf* expression correlated with albuminuria and glomerular basement thickness [7]. In addition to the reported positive association between C*tgf* expression and extracellular matrix proteins, recent data derived from a *Col1a2* promoter insertional transgenic mice model directly link increased C*tgf* overexpression with fibrosis in multiple organs including kidney [8]. It is also worth noting that, whereas a polymorphism in *ctgf* promoter has been linked with systemic sclerosis [9]–[11] and infectious hepatic fibrosis [12], these observations have not been consistent [13]–[15].

There is emerging evidence that link changes in plasma and urinary CTGF levels to diabetic nephropathy. Plasma CTGF levels are elevated in persons with type 1 DM and plasma CTGF levels have been correlated with proteinuria and creatinine clearance [16]. Likewise, in a study of persons with type 1 DM, urinary CTGF levels were elevated in those with diabetic nephropathy and correlated with albumin excretion rate (AER) and glomerular filtration rate (GFR) [17]. In addition, an evaluation of glomerular CTGF protein in biopsies from type 1 diabetic patients revealed increased CTGF expression as disease progressed from incipient to advanced nephropathy [18]. In STZ-induced diabetic and ob/ob diabetic mice, glomerular C*tgf* expression also mirrored development of nephropathy and correlated with urinary CTGF protein [19]. These observations strongly suggest a pathologic role for CTGF in diabetic nephropathy and, as well, a recent report [20] provides putative mechanistic insights into the manner in CTGF may affect matrix biology in the setting of hyperglycemia and/or diabetes mellitus. The current study examined the effect of systemic *Ctgf* expression on kidney function, albuminuria and susceptibility to diabetic kidney disease

## Materials and Methods

### Chemicals and Reagents

Dulbecco's modified Eagle's medium (DMEM), penicillin-Streptomycin and trypsin (0.05%)-EDTA solutions were purchased from Invitrogen (Carlsbad, CA, USA). Restrictions enzymes were from New England Biolabs (Ipswich, MA, USA), while Effectene transfection reagent was procured from Qiagen (Valencia, CA, USA). Except where stated, all other chemicals were purchased from Sigma-Aldrich (St. Louis, MO, USA). Kits for determination of plasma triglyceride (product No: 461-08992), cholesterol (product No: 439-17501) and glucose (product no: 439-90901) were sourced from Wako Diagnostics (Richmond, VA, USA). Blood urea nitrogen (BUN; product no: 700620) was measured with assay kits from Cayman Chemical (Ann Arbor, MI, USA).

### Animals

**Figure 1:**
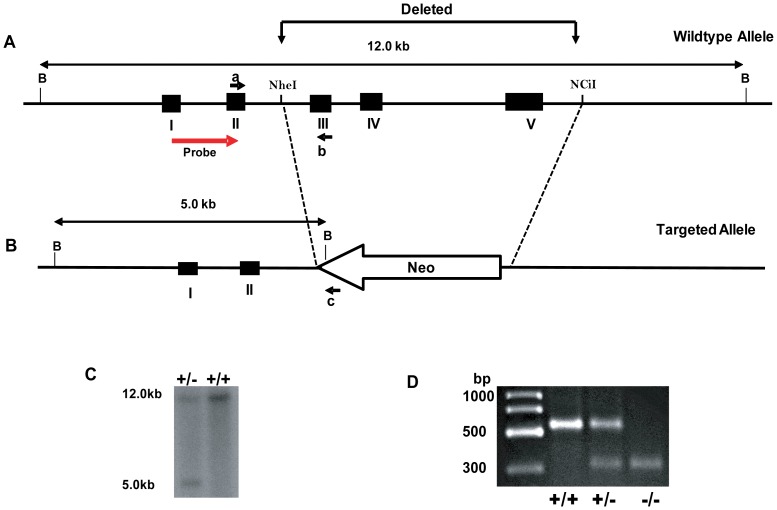
Generation of mice with disrupted *Ctgf* gene. Depiction of wildtype allele is represented in Panel **A**. I,II, III, IV and V represents exons 1 to 5 of *Ctgf* gene. The arrow straddling exons I and II represents 5′ probe. B (for *Bam*HI); NheI and NciI represent restriction enzyme sites. Targeted allele (Panel **B**) demonstrating loss of exons III to V following recombination in ES cells. *Neo* represents neomycin resistance gene. Southern blot analysis of genomic DNA is shown in Panel **C**. The targeted allele was identified by 5′probe that hybridizes to a 5 kb fragment in the heterozygous and 12 kb fragment in the wildtype. PCR of genomic DNA from MEF is represented in Panel **D**. In panel A, **a**,**b** and **c** indicate location of primers used for PCR, whereas B denotes restriction site for *Bam*H1. Primers **a** and **b** gives a 550 bp endogenous product, while primers **a** and **c** gives a 320 bp targeted product as indicated in panel D.

All animal experiments were carried out after approval from, and in accordance with, the Institutional Animal Care and Use Committees at UT Southwestern Medical Center at Dallas (Animal Protocol Number 0993-06-02-1) and the University of North Carolina at chapel Hill (Protocol Numbers 07-228 and 05-046). 129S6 wildtype mice were purchased from Taconic (Hudson, NY, USA), while C57BL/6 wildtype mice were from Charles River Laboratories (Wilmington, MA, USA). CTGF knockout mice were generated through gene targeting in 129S6 mouse embryonic stem (ES) cells by replacing exons 3 to 5 of endogenous *ctgf* with a neomycin resistance (*neo*) cassette (figure 1). Mice carrying the mutated *ctgf* gene were subsequently generated by standard techniques [21] and maintained on a 129S6 inbred background. Mice were kept on 12-hour light-dark cycle at ambient temperatures of 68°F to 72°F and had free access to water and food (regular rodent diet [diet# 2014, Harlan Teklad, Madison, WI]).

### Intraperitoneal (IP) STZ injections

Mice (n = 10/group) were rendered diabetic by employing a low dose STZ protocol [22]. Briefly, mice were injected with freshly prepared STZ dissolved in 0.05M citrate buffer (0.05 ml; pH 4.5) at a dose of 40 µg/g BW on five consecutive days. Control animals (n = 9) were injected with citrate buffer only. Mice were followed for up to 4 months following the induction of diabetes.

### Physiologic/Metabolic Studies

Mice were placed in metabolic cages (Hatteras Instruments, Cary NC) overnight with free access to food and water. Body weight, food intake, water consumption and urine volume were recorded before and after the study interval. Following induction of anesthesia with Avertin (2,2,2-tribromoethanol, 1.25%), blood was obtained from the retro-orbital sinus of each mouse using a microcapillary tube (0.075 ml; Fisher Scientific). Urine albumin was determined using commercially available kits (Exocell Inc. PA). Albumin excretion rate, AER (µg/24 hours) was estimated from the urine albumin determined by ELISA. Creatinine was determined by capillary electrophoresis (UT Southwestern O'Brien Core Lab) using a P/ACE MDQ Capillary Electrophoresis System (Beckman Coulter Inc) as previously reported [23], [24]. Prior to capillary electrophoresis, plasma specimen (0.1 ml) were treated with trichloroacetic acid (TCA; 5% [v/v]; 0.1 ml) and precipitate proteins were removed by centrifugation (3000×g/5 minutes) [24].

### Histology

Following euthanasia, carcasses were initially perfused (perfusion pressure 70 and 100 mm Hg) with phosphate-buffered saline (PBS, 10 mls over 1 min via gravity feed)) solution and subsequently with paraformaldehyde (PFA [4% v/v]; 50 mls over 4 minutes). Fixed kidney sections were dehydrated, embedded in paraffin, and 3–4 µM sections were made for histologic analyses. Specimens were analyzed by light microscopy to assess glomerular and renal tubular histology. Mesangial matrix expansion was estimated by a semi-quantitative method [25]. Changes observed on PAS-stained sections were assigned a value of 1 to 4, in a blinded fashion by 2 observers, depending on the number of glomeruli manifesting matrix expansion. Accordingly values were assigned as follows: score 0, a normal glomerulus; score 1, increased mesangial matrix of up to 25% of glomerular tuft; score 2, mesangial expansion of 25 to 50% of glomerular tuft; sore 3, mesangial expansion of 50 to 75%; and score 4, mesangial expansion of >75% of the tuft.

### Immunohistochemistry

Paraformaldehyde-fixed paraffin-embedded tissues were cut into 4-µm sections. The slides were heated (30 minutes, 56°C), then deparaffinized (three washes; 5 minutes each) in HistoClear (National Diagnostics, Atlanta, GA, USA) and rehydrated through a graded alcohol series (100%, 95%, and 70% washes for 5 minutes each wash). For antigen retrieval, the slides were autoclaved in 10 mM sodium citrate buffer. Sections were pre-incubated in PBS and then blocked in PBS containing 5% bovine serum albumin (BSA) and 1% goat serum (30 minutes at room temperature). The sections were incubated overnight at 4°C with primary antibodies against insulin (sc-7838;1∶100; Santa Cruz Biotechnologies) and glucagon (sc-13091; 1∶100; Santa Cruz Biotechnologies). The sections were rinsed with PBS and incubated with IgG secondary antibody conjugated to Alexa Fluor 488 or Alexa Fluor 555 (Molecular Probes; 1∶400) for 1 h at room temperature, and counterstained with 4′-6-diamidino-2-phenylindole (DAPI, Boehringer Mannheim; 1∶5000). Fluorescent signals were visualized by using a Zeiss Axioplan 2 Imaging System (Carl Zeiss MicroImaging Inc., Thornwood, NY, USA).

### Cell Isolation and Culture

Mouse embryonic fibroblasts (MEF) were prepared from ED12.5 to ED14.5 embryos [26]. Stable lines of spontaneously immortalized MEF were generated by serial splitting as described [27]. Primary mouse mesangial cells (MMC) were prepared from 2 month old male wildtype 129S6 mice by the sieving method [28], [29]. Cells between passage 8 and 12 were used in these studies. Cells were initially plated on plastic culture dishes and maintained (37°C/5%CO_2_) in DMEM/10% fetal bovine serum (FBS) supplemented with penicillin (100 IU/ml)/Streptomycin (100 µg/ml). Twenty-four hours later, medium was changed to one containing 0.5% FBS with and without high glucose (25 mM), or inhibitors for ERK, p38MAPK and JNK. Cells were cultured for an additional 48 hours prior to harvesting.

### CTGF and Thrombospondin 1 promoter-Luciferase Reporter Constructs

To generate a mouse thrombospondin 1 (TSP1) gene promoter, a 1.3 kb S*ac1*/H*indIII* fragment encompassing the transcriptional start site and upstream promoter region was synthesized as above using primers 5′-ATAGAGCTCCCCTGATTCTGCCAAGATCCT-3′ and 5′-TAAAAGCTTATGAGGCTGGCTGACTCCAG-3′. Following confirmation of sequence fidelity (UT Southwestern sequencing Core), the promoter fragment was ligated into a luciferase reporter vector (pGL3 Basic; Promega Corporation). The resultant *Tsp1*-luciferase construct (pTsp-Luc) was used for transient expression in wild-type primary MMC or in (MEF) respectively.

### Transfection

MMC (1.5×10^5^ cells/well) or MEF were plated onto six-well plastic plates (Falcon #353224, Becton Dickinson, NJ, USA), and transfection was carried out 24 hours later using Effectene (QIAGEN) according to the manufacturer's specifications. Briefly, cells (70–80% confluent) were co-transfected with 0.25 µg of pTsp-Luc, and 0.05 mg of pCMVβgal and then cultured for 18 h in DMEM containing FBS (20%) and 5.6 mmol/l D-glucose. With the use of CMV promoter-driven green fluorescent protein construct, the efficiency of transfection was estimates to 35–40%. Subsequently, the media was changed to DMEM with 0.5% FBS and glucose (5.6 or 25 mmol/l) and MMC were incubated for an additional 48 hours prior to harvesting.

### ECM protein Expression in MEF

ECM expression was analyzed with a commercially available expression profiling kit (product #PM-012B, SA Biosciences). Briefly, cells (1×10^6^/100 mm plate) were seeded in DMEM containing FBS (10%) and D-glucose (5.6 mmol/L) for 24 hours. Subsequently, the media was changed to DMEM with 0.5% FBS and glucose (5.6 or 25 mmoles/L) for 48 hours. At the end of this period, cells were harvested and total RNA was isolated by standard methods [30]. Gene expression was subsequently analyzed by RT-PCR per manufacturer's specifications.

### Western Blot Analysis

This was performed as we have previously described [31], [32]. Subsequent to retrieval of kidney from euthanized animals (0.5 mls 2.5% Avertin), protein was extracted from kidney cortical slices suspended in MPER protein extraction reagent (product No. 78503, Pierce, Rockford, IL) in the presence of protease inhibitors (product No. P8340, Sigma-Aldrich) using a homogenizer (Kinematica, Luzen, Switzerland). Protein extracts were separated using 12% SDS-polyacrylamide gel electrophoresis under reducing conditions. The separated proteins were transferred to nitrocellulose membranes and incubated with commercially available rabbit polyclonal (primary) antibodies to either CTGF (GTX25097, GeneTex, San Antonio, TX), Nephrin (sc-28192, Santa Cruz Biotechnology Inc, Santa Cruz, CA, USA), thrombospondin 1 (AB2962; Abcam, Cambridge, MA) or fibronectin (sc-9068, Santa Cruz Biotechnology Inc, Santa Cruz, CA, USA). Subsequently, CTGF, thrombospondin 1 and fibronectin were identified with peroxidase conjugated anti-rabbit secondary antibody, and ECL Western blotting analysis system (Amersham). Autograms were quantified by scanning densitometry.

### RT-PCR

Total RNA was extracted from MEF and kidney cortex using TRIzol reagent (Invitrogen, Carlsbad, CA) in accordance with the manufacturer's protocol. Gene expressions were determined using real-time quantitative reverse transcription PCR (RT-PCR) (Applied Biosystems, Foster City, California, USA) with β-actin as the reference gene in each reaction [33].

### Statistical Analysis

All statistical analyses were performed using the JMP statistical Software (SAS Institute, Cary, NC). Values are reported as mean ± standard deviation. Comparison between groups was assessed with ANOVA and the Tukey test; differences were considered significant at p<0.05.

## Results

### Characteristic of CTGF-Knockout Line

**Figure 2:**
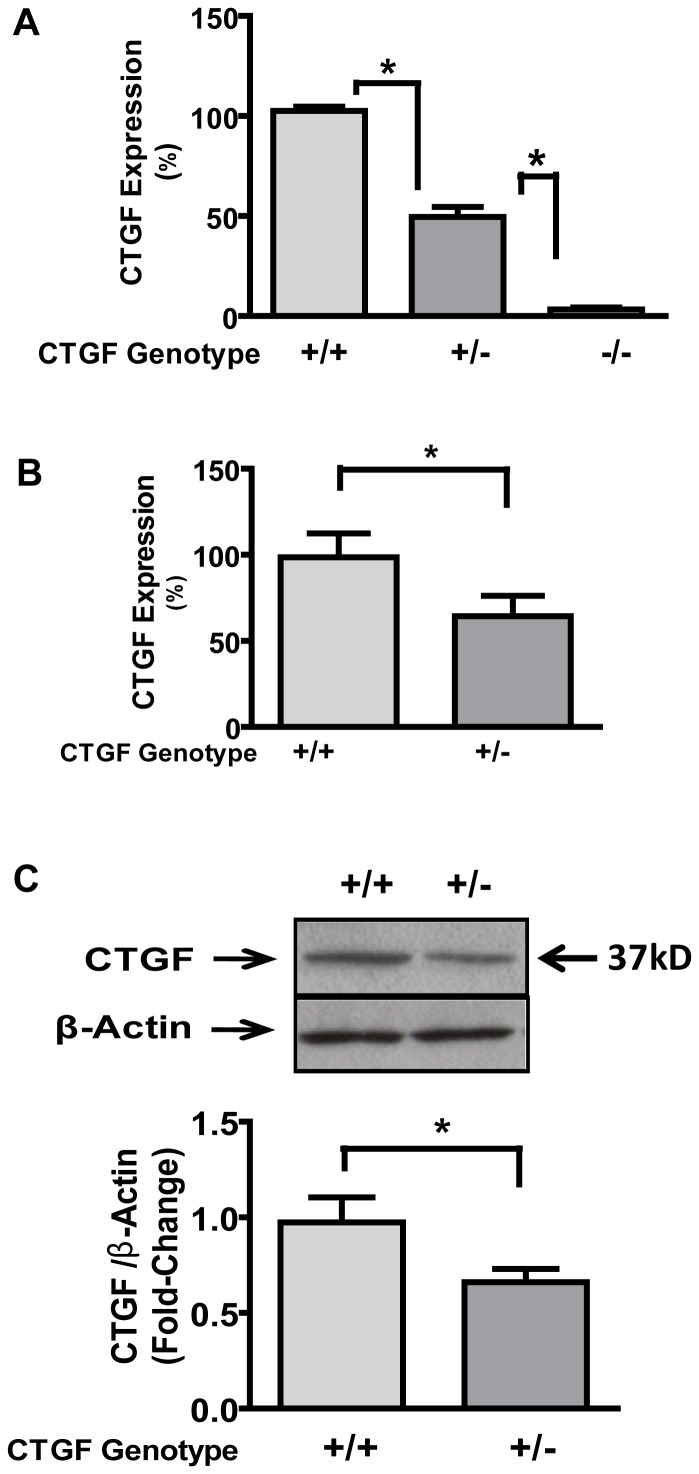
CTGF mRNA levels in kidneys from newborn (A) and 1-month old mice (B). Total RNA was isolated and expression was determined by qRT-PCR as described in methods. Panel **C** depicts CTGF protein levels in kidney from wildtype and heterozygous adult mice. Protein was determined by Western blotting analysis. *p<0.05; n = 6.

CTGF gene knockout (CTGF**^−/−^**) mice were generated by replacing exons 3 to 5 of C*tgf* with bacterial neomycin resistance (*Neo*) through gene-targeting in 129 SvEv-Tac derived embryonic stem (ES) cells as described in the methods (above). Total RNA from tail snips and mice embryonic fibroblasts [30] was used for RT-PCR. Quantitative RT-PCR (qRT-PCR) performed using total RNA from tail and kidney cortex revealed that Ctgf expression in heterozygous was approximately 50% of the level in wildtype mice (Figure 2A and B). In kidney specimen from heterozygous mice, the level of CTGF protein was about 60% of that in control (wildtype) littermates (Figure 2C).

Homozygous gene-disrupted (CTGF**^−/−^**) mice die within 24 hours of birth, but heterozygous offspring survive and thrive normally. Fertility, growth and development features, relevant physiologic and hematologic parameters of heterozygous mice (CTGF**^+/−^**) are not significantly different from wildtype (CTGF**^+/+^**) littermates (Table 1). Several of these findings are similar to that described for *Ctgf* disruption by other investigators [34]. However, blood pressure was lower in heterozygous mice compared with wildtype littermates (table 1).

**Table 1:** Baseline clinical characteristics of wildtype and CTGF heterozygous mice.

	Wildtype	Heteozygous
	(+/+)	(+/−)
Number of Animals (n)	8	8
Glucose (mmol/L)	6.9±1.7	7.5±0.8
Triglyceride (mmol/L)	0.4±0.2	0.3±0.1
Cholesterol (mmol/L)	3.4±0.4	3.1±0.5
Creatinine (µmol/L)	5.8±1.2	6.0±1.1
BUN (mmol/L)	12.1±1.0	11.5±2.8
Hematocrit (%)	49±1.5	50.5±1.1
PT (seconds)	12.1±0.2	11.9±0.
Blood Pressure (Systolic, mmHg)	112±4	*101±3

**Note:** Knockout mice die within 24 hours after birth. Values represent mean ± SD;

* p<0.05.

### Proteinuria is attenuated in diabetic ctgf heterozygous mice

Given the putative role of CTGF in the response to elevated levels of extracellular glucose and diabetes mellitus, we performed studies to determine the effect of streptozotocin (STZ)-induced diabetes mellitus on plasma creatinine and albuminuria in CTGF**^+/−^** mice. Mice were rendered diabetic with the low dose STZ protocol [22], while control animals received citrate buffer (vehicle). Two mice from the STZ group and one from the control (buffer only) group died over the course of the study. Data from these animals have not been included in the analysis. Diabetic CTGF**^+/+^** and CTGF**^+/−^** mice consumed more food and water and produced more urine than their non-diabetic counterparts (Figure S1). However, there were no differences between diabetic mice of both genotype with respect to food and water consumption or urine output. Likewise, there were no significant differences in body weight amongst the 4 groups of animals (Figure S1).

**Figure 3:**
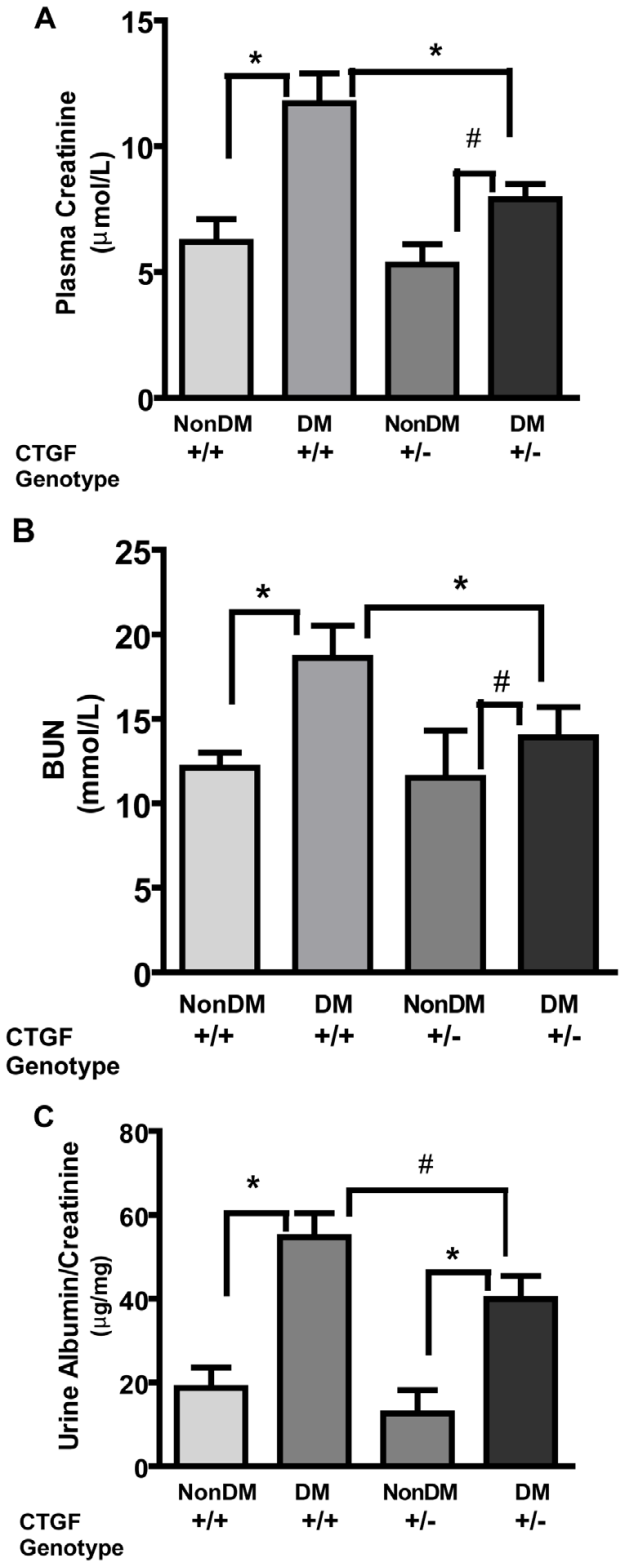
Plasma creatinine (A), urea nitrogen (BUN, [B]) and urinary albumin to creatinine ratio (C) are increased in diabetic wildtype (+/+) and CTGF heterozygous (+/−) mice after 4 months of STZ-induced diabetes mellitus. For all parameters, the increase for CTGF heterozygous animals was significantly less when compared to that for wildtype littermates. Values represent the mean ± standard deviation; *p<0.01; #p<0.05; NS – not significant; n = 8.

Plasma glucose increased after the STZ treatment, but was not different between the CTGF**^+/+^** and CTGF**^+/−^** mice throughout the study (table 2). Although CTGF**^+/+^** animals had plasma glucose levels similar to CTGF**^+/−^** mice (28.2±3.3 vs 27.0±3.1 mmol/L), the latter group had lower plasma creatinine levels (7.9±0.6 vs 11.7±1.2 µmoles/L, p<0.01; figure 3A). Likewise, blood urea nitrogen (BUN) and urinary albumin/creatinine ratio were significantly attenuated in diabetic CTGF**^+/−^** when compared with their diabetic CTGF**^+/+^** group (figure 3B and C). Whereas plasma cholesterol was unaffected by diabetes, plasma triglyceride was significantly lower in diabetic CTGF**^+/−^** mice compared with CTGF**^+/+^** animals (table 2). Histologic and immunohistochemical analysis of endocrine and exocrine pancreas from non-diabetic wildtype and heterozygous animals (Figure S2) did not reveal any obvious basal differences between the groups that may account for observed variation in glucose or triglyceride disposal.

**Table 2:** Clinical characteristics of diabetic wildtype and CTGF heterozygous mice.

	Wildtype	Heteozygous	P
	(+/+)	(+/−)	
Number of Animals (n)	8	8	
Glucose (mmol/L)	28.2±3.3	27.0±3.1	NS
Triglyceride (mmol/L)	0.7±0.2	0.5±0.1	<0.05
Cholesterol (mmol/L)	3.6±0.4	3.9±0.6	NS
Creatinine (µmol/L)	11.7±1.2	7.9±0.6	<0.01
BUN (mmol/L)	18.6±1.9	14±1.8	<0.01
Hematocrit (%)	48±2.5	51.3±3.1	NS
Blood Pressure (mmHg)	109±14	104±8	NS

**Note:** Values represent mean ± SD; NS – not significant.

### CTGF influences Mesangial Matrix accumulation

**Figure 4:**
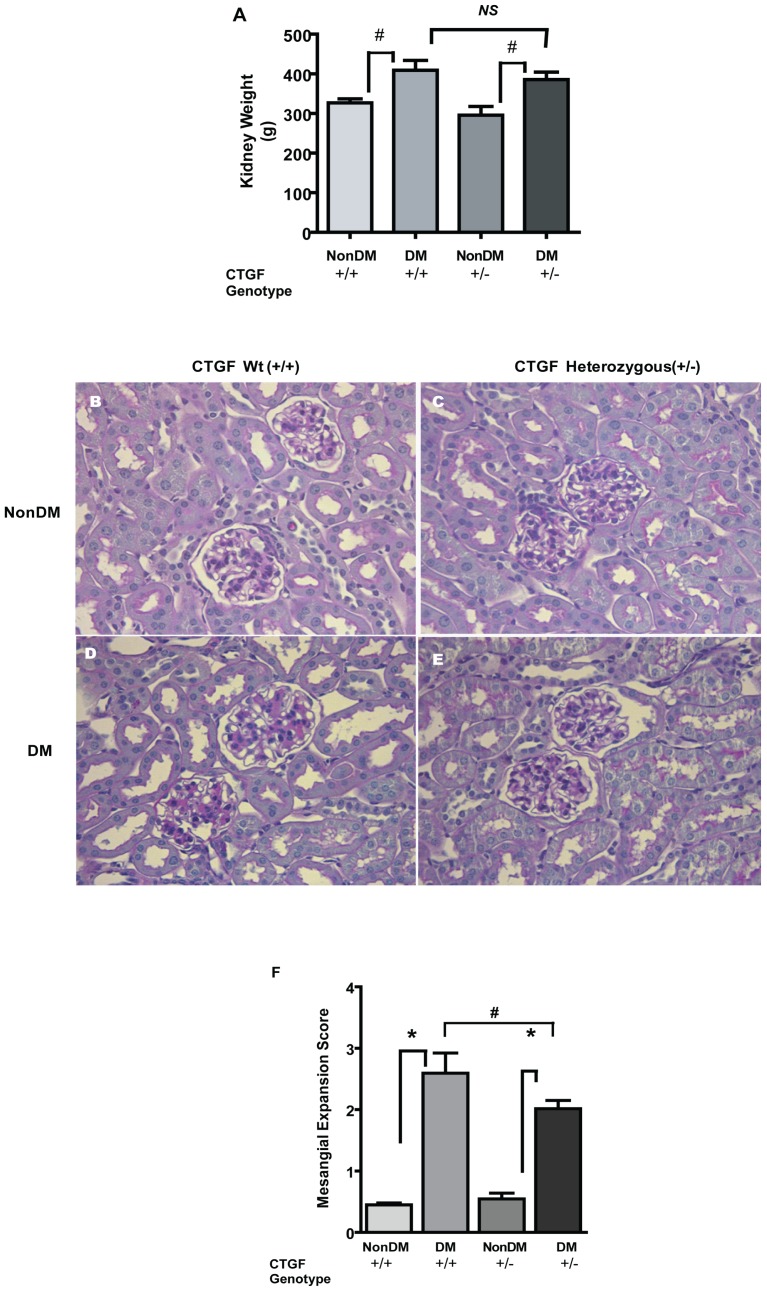
Kidney/body weight ratios are increased in diabetic mice, without a significant difference between diabetic wildtype and heterozygous mice (Panel A). Representative kidney sections from non-diabetic wildtype [**B**] and CTGF heterozygous [**C**] and diabetic wildtype mouse [**D**] and diabetic CTGF heterozygous littermates [**E**] reveal increase in mesangial matrix is attenuated in diabetic CTGF heterozygous mice as compared to that observed in kidneys from wildtype animals. Panel **F** depicts estimated mesangial expansion score for the various groups. Kidneys were perfusion-fixed with PFA (4%), embedded in paraffin and sectioned (4 µm) followed by PAS staining. Original magnification ×200; *p<0.01; #p<0.05.

The data in the previous section suggest that albuminuria is less severe in diabetic CTGF**^+/−^** mice than in CTGF**^+/+^** animals, suggesting that reduced C*tgf* expression may offer protection against proteinuric kidney disease in this model. As CTGF is implicated in ECM production, the effect on kidney size and glomerular matrix was assessed. As expected, diabetes lead to increases in kidney mass in both CTGF**^+/+^** and CTGF**^+/−^** animals, but the difference between the two groups of diabetic animals was not significant (figure 4A). We observed reduced mesangial expansion in diabetic CTGF**^+/−^** relative to their CTGF**^+/+^** littermates (figure 4B to E). The latter finding was further bolstered by the observation of lower mesangial expansion scores in diabetic CTGF**^+/−^** when compared with diabetic CTGF**^+/+^** mice (Figure 4F). Simplification of tubular epithelial cells was observed in kidney sections from diabetic animals (Figure 4d). Masson-Trichrome stained sections showed mild interstitial fibrosis, but this was not markedly different from that observed in non-diabetic wildtype and heterozygous animals (Figure S3).

### High-glucose induced expression of extracellular matrix proteins is dependent on CTGF

CTGF has been shown to mediate expression of extracellular matrix (ECM) proteins in response to various external perturbations [6], [35]. To further delineate the role of CTGF role in the cellular response to high ambient glucose levels, we initially examined the levels of known extracellular matrix protein by mice embryonic fibroblasts (MEF). Matrix accumulation in diabetic and other proteinuric kidney diseases may be mediated by resident fibroblast and mesangial cells. Some studies suggest that cells with phenotypes of mesangial cells and fibroblasts (fibroblast/myofibroblast) are the cell type most responsible for matrix accumulation that is observed in proteinuric kidney diseases, like diabetes [36], [37]. Fibroblast cell lines were selected for study to reflect properties of both cell types (mesangial cells and fibroblasts). Accordingly, MEF were isolated from CTGF**^+/+^** and CTGF**^+/−^** embryos. Stable lines of CTGF**^+/+^** and CTGF**^+/−^** MEFs were exposed to physiologic or high glucose (25 mmoles/L) for 48 hours and extracted total RNA was used to assess the impact on extracellular matrix proteins expression using a commercially available expression panel.

**Figure 5:**
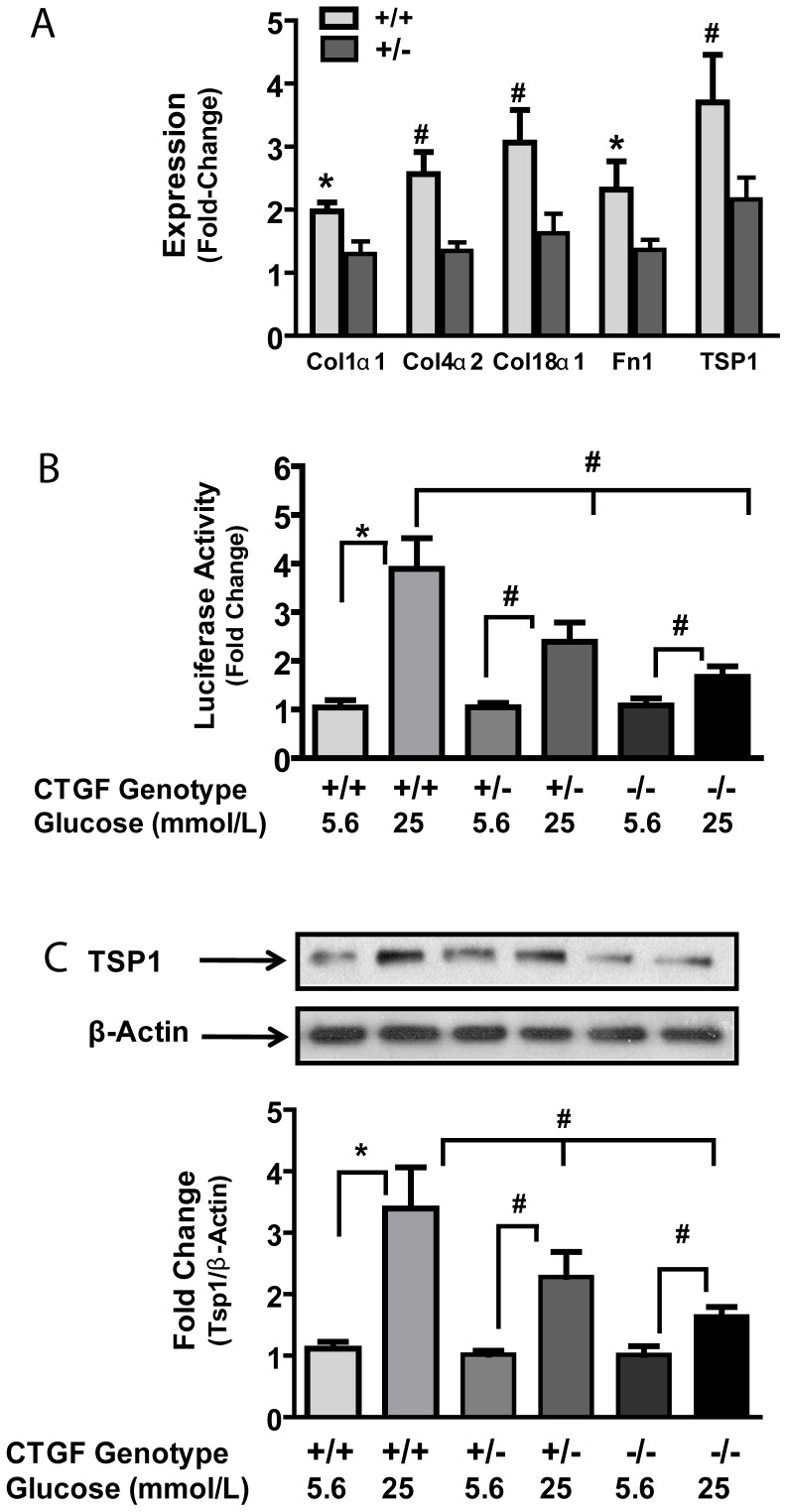
ECM expression of selected genes in wildtype (+/+) and heterozygous (+/−) MEF cell lines exposed to high glucose (25 mM) for 48 hours (A). The changes have been normalized to that observed in physiologic glucose (5.6 mM). TSP1 promoter expression (**B**) and protein (**C**) are increased by high glucose in wild type MEF and attenuated in *ctgf*-heterozygous and knockout MEFs. ECM expression array studies were done in duplicate and repeated twice. All other experiments were performed in triplicates and repeated at least 3 times. Values are mean +/− SD. *p<0.05; #P<0.01. Abbreviations: wildtype +/+; heterozygous +/−; knockout −/−; Col1α1 – alpha 1 collagen type I; Col4α2- alpha 2 collagen type IV; Fn1 – fibronectin 1; Col18α1 – alpha 1 collagen type XVIII; TSP1 – thrombospondin 1.

In CTGF**^+/+^** cells, high ambient glucose induces a 2- to 3-fold increase in expression of several ECM proteins including α1-collagen 1 (Col1α1), α2-collagen IV (Col IVα2), α1-collagen XVIII (Col18α1), fibronectin (Fn) and thrombospondin 1 (Tsp-1) (Figure 5A and table S1). The observation for Col18α1 has not being previously reported, but the effects of high glucose on the other ECM protein expression are consistent with earlier reports for other cell lines [6], [35]. In contrast, in CTGF**^+/−^** cells exposed to high ambient glucose, expression of the aforementioned ECM proteins was attenuated (figure 5A). The data further support a role of CTGF in mediating the cellular response to high extracellular glucose levels that accompanies diabetes mellitus.

**Figure 6:**
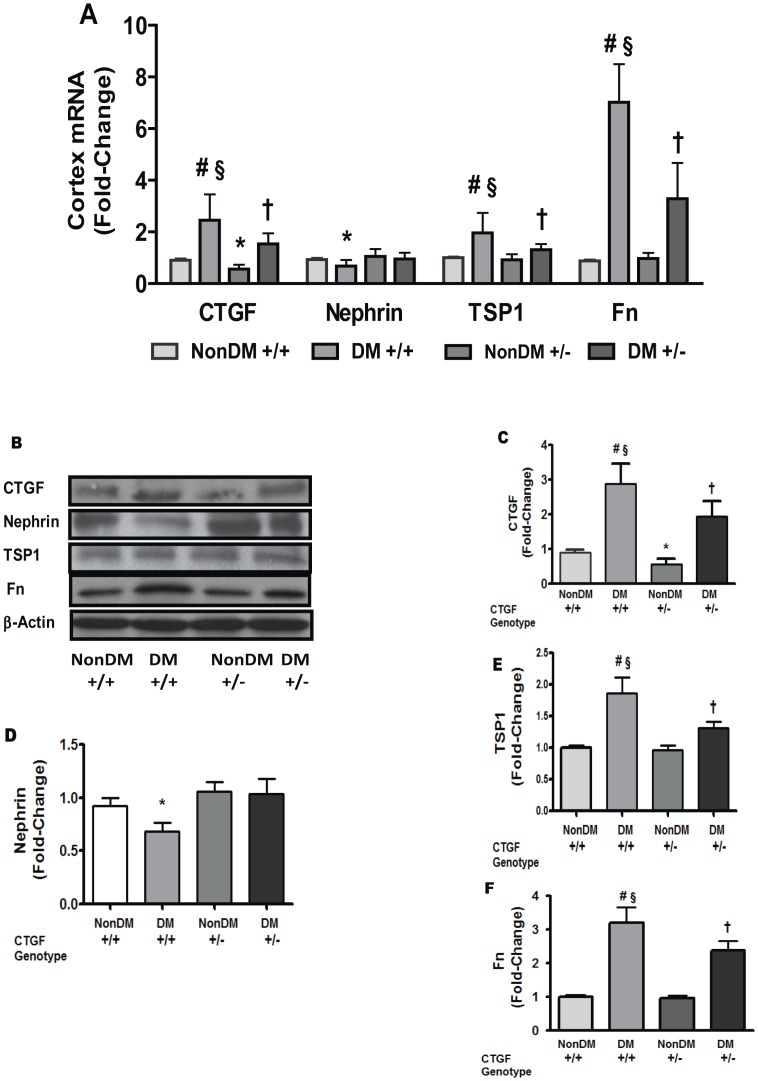
Expression of CTGF, TSP1 and Fibronectin (Fn) is increased, while nephrin is decreased in kidney cortices from diabetic wildtype mice, but attenuated in diabetic heterozygous animals (A). Similar findings were observed for CTGF, Nephrin, TSP1 and Fn proteins (**B to F**). Total RNA and protein were extracted from PBS-perfused kidney tissues; mRNA levels were determined by qRT-PCR whilst protein was assessed by Western Blotting Analysis. Values represent mean ± SD, n = 4. *p<0.05 vs -NonDM+/+; **#**p<0.02 vs NonDM+/+; †P<0.05 vs NonDM+/−; §p<0.05 vs DM+/−.

*Tsp1* is induced in cells cultured under high glucose conditions [38]. In humans with type 1diabetes, progressive increases in CTGF and TSP1 accompanies the development and progression of diabetic kidney disease; in mesangial cells, CTGF mediated glucose-induced fibronectin synthesis [18]. However the effect of CTGF on TSP1 is ill-defined. Given the observation that Tsp1 expression was attenuated in C*tgf* heterozygous MEF, we chose to corroborate the finding of the expression array by initially studying activation of TSP1 promoter-luciferase reporter and secondly by assessing TSP1 protein levels in cells cultured under physiologic and high glucose conditions. As indicated in figure 5 B, CTGF**^+/+^** MEF exposed to high glucose show an increase in TSP1 promoter activity suggesting transcriptional regulation. In CTGF**^+/−^** and CTGF^−/−^ MEFs, there was attenuation of *Tsp*1 promoter activity further supporting a role for CTGF in *Tsp*1 promoter activity. Similarly, TSP1 protein levels were increased by high glucose in CTGF**^+/+^** MEFs and attenuated in CTGF**^+/−^** and CTGF^−/−^ MEFs (figure 5 C). However, for TSP1 protein and promoter activity, the difference between CTGF**^+/−^** and CTGF^−/−^ MEFs was not significant. In line with the observations in MEFs, we observed that both CTGF and TSP1 messages were increased in kidney cortex from diabetic CTGF**^+/+^** compared with kidney derived from diabetic CTGF**^+/−^** mice (figure 6). In contrast, nephrin expression was decreased in diabetic CTGF**^+/+^** mice but unchanged in diabetic CTGF**^+/−^** mice (figure 6).

## Discussion

A significant consequence of abnormal response to injury is increased expression of CTGF. CTGF mediates induction of genes for various extracellular proteins including thrombospondin I (TSP1) in bleomycin induced pulmonary fibrosis, plasminogen activator inhibitor type 1 (PAI-1) and collagen I, III and IV in rat kidney fibroblasts [39]–[41]. In addition, there is emerging evidence that link changes in plasma and urinary CTGF levels to diabetic nephropathy [16]–[19]. Recent observations suggest that ctgf overexpression is sufficient to mediate fibrosis [8]. Accordingly, we sought to determine the effect of systemic *ctgf* expression on kidney function, albuminuria and susceptibility to diabetic kidney disease in an animal model with diminished *Ctgf*.

The initial observation, that homozygous gene-disrupted mice die within 24 hours of birth, is similar to that published by other investigators in which mice on a mixed C57Bl/6 – 129S6 background were studied [34]. This implies that the early post-natal demise, that we and other have observed, reflects an important role for CTGF in perinatal survival which is independent of background strain of the mice. On the other hand, several parameters (blood pressure, lipids, creatinine, BUN) which have been determined for CTGF**^+/−^** mice in the current study (Table 1) have not been previously reported. In the current study, parameters that have been previously reported are not different from those of CTGF**^+/+^** littermates, suggesting that *ctgf* heterozygosity did not appear to exert significant influence in the non-diabetic state.

Systemic blood pressure was lower in heterozygous compared with wildtype mice. However, the reason(s) for this difference in blood pressure between wild type mice and those that are heterozygous for *ctgf* are unclear and have not been previously reported. Likewise, the impact of CTGF on system blood pressure is unknown. RAAS blockade, that classically lead to better blood pressure control, improvement in proteinuria and renal protection, has been associated with reduced CTGF expression [42], [43]. However, this effect is related to upstream influence of RAAS on CTGF, rather than inherent effect of CTGF. Nonetheless, given that CTGF can influence extracellular matrix protein like collagen and fibronectin, it is conceivable that this may in turn affect blood vessel, peripheral resistance and systemic blood pressure.

We observed that in STZ-diabetic mice, *Ctgf* heterozygosity attenuates increases in plasma creatinine levels, albumin excretion and mesangial matrix expansion. Using a related mouse model [34], *ctgf* heterozygosity has also been found to be protective with respect to albuminuria and proteinuria in animals with diabetes [20]. These observations, that a roughly 50% reduction in the *Ctgf* expression attenuates changes in kidney function, provide a possible causative link between progressive kidney disease (manifested by albuminuria and/or urinary albumin/creatinine ratio) and *Ctgf* expression. Plasma creatinine was not reported in the study by Nguyen et al [20], probably because of the difficulty of measuring this parameter in mice plasma. In the current study, utilizing capillary electrophoresis, we have found that in STZ diabetic mice changes in plasma creatinine occur in concert with albuminuria and that *ctgf* heterozygosity mitigates these changes. In line with these latter findings, we also observed attenuation of mesangial expansion in the diabetic CTGF**^+/−^** mice compared with diabetic CTGF**^+/+^** animals. Attenuation of mesangial expansion in CTGF**^+/−^** mice is consistent with observations highlighted by another report [20] and further supports a role for CTGF in the production of ECM proteins in response to high extracellular glucose. Simplification of tubular epithelial cells was observed in kidney sections from diabetic animals (Figure 4d). Masson-Trichrome stained sections showed mild interstitial fibrosis, but this was not markedly different from that observed in non-diabetic wildtype and heterozygous animals (Figure 3). These observations are in accord with that reported for genetic diabetic models studied from age 8–10 weeks for 4 months [44].

Hypertriglyceridemia is a feature of diabetes mellitus [45]. In the current study, plasma triglyceride levels were similar in wildtype and heterozygous animals (table 1). However, in the diabetic state there was a modest, but significant increase in plasma triglyceride in wildtype compared to heterozygous mice (table 2). The reason(s) for difference between the two groups of animals in the diabetic state is not known. Plasma triglyceride levels are determined by production and clearance of TG containing lipoproteins [46]. Pancreatic lipase participates in clearance of triglyceride carried in lipoprotein moieties (primarily chylomicrons and very low density lipoproteins) [47], [48], but the impact of CTGF on lipase production, release or activity is unknown. CTGF may be important in pancreatic islet development [49] and may have an influence on pancreatic cancer development [50]. However, histology examination (H&E and IHC studies; Figure 2) did not reveal differences between the two groups. Nonetheless, because of a putative role for CTGF in pancreatic development, it is possible that CTGF may, through effects on exocrine and endocrine pancreas, impact triglyceride-rich lipoprotein metabolism.

Overall, difference between the current study and that previously reported [20] may be due to genetic background. Indeed, genetic background has a significant impact on diabetic phenotype in mice [44], [51]. In particular 129 mice manifest greater degree of proteinuria and mesangial matrix expansion compared with C57BL/6. Thus, our observation that the phenotype described in this study was more robust than that reported by Nguyen et. al. [20] likely reflects strain differences. We used 129 line while a mixed C57BL/6-129 was used by Nguyen et. al.

To further study the role of CTGF in ECM response to high extracellular glucose, we examined the effect of *Ctgf* expression on ECM from cells cultured in high glucose. Stable MEFs were generated from embryos (see ‘Methods’) and expression of ECM proteins was determined following exposure to physiologic or high glucose levels. Amongst the genes studied, col1α1, col4α2, Col18α1 and fibronectin 1 (Fn1) and thrombospondin 1 (TSP1) were all increased in CTGF**^+/+^** cells. In this series of experiments we observed that reduced *ctgf* expression attenuated expression of several ECM proteins (figure 5A and Table S1). Col18α1 expression in diabetic kidney disease has not been previously reported. However, the findings with respect to col4α2, Col18α1 and TSP1 are significant because of their potential role in fibrosis and anti-angiogenesis. Procollagen IV and procollagen XVIII are parent molecules of anti-angiogenic factors tumstatin and endostatin respectively, while TSP1 has potent anti-angiogenic and pro-fibrotic properties [52], [53]. The observation that collagen XVIII is upregulated by hyperglycemia was unexpected. Collagen XVIII has been identified in basement membrane of vascular and epithelial cells [54]. In this regard, collagen XVIII may be protective in immune-mediated glomerulonephritis in that collagen XVIII deficient mice develop more severe glomerular disease than wildtype littermate [55]. Collagen XVIII may also affect podocytes in that variable foot process effacement has been described in collagen XVIII knockout mice [56]. On the other hand, a role for collagen XVIII in the pathogenesis of diabetic kidney disease remains unclear. Although reduction in *ctgf* expression may not protect again fibrosis that occurs in severe injury [57], overall our observations implicate *ctgf* expression as a mediator of ECM protein expression in response to high-glucose.

TSP1 is induced in cells cultured under high glucose conditions [38] and CTGF may influence TSP1 levels in cultured renal interstitial fibroblasts [58]. In this context, some studies suggest that TSP1 is key regular is transforming growth factor beta (TGFβ) activity and that glucose-induced TGFβ bioactivity is mediated by TSP1 [59]. In humans with type 1 diabetes mellitus, progressive increases in CTGF and TSP1 accompany the development and progression of diabetic kidney disease and, in mesangial cells, CTGF mediates glucose-induced fibronectin synthesis [18]. In line with these earlier observations, we found that CTGF**^+/+^** MEF exposed to high glucose (25 mM) manifested a significant increase in expression of ECM proteins including TSP1 (figure 5, panel A). The observation for TSP1 was confirmed with TSP1 promoter-luciferase reporter system (figure 5, panel C) and western blotting analysis (figure 5, panel B). Our observation of increased TSP1 expression in kidney (figure 6) supports these *in vitro* observations. The associated observation that *ctgf* expression was attenuated in kidney derived from CTGF**^+/−^** mice (figure 6) is in line with previously published data [20]. Likewise, in diabetes, a reduction in nephrin expression and commitment increase in *Ctgf* expression have been previously reported [60]. Our current observations support these recent findings.

Reduced *Ctgf* expression in heterozygous cells attenuated high glucose-induced TSP-1 expression. However, in ctgf-null MEFs, the effect of high glucose on TSP1 expression was not abolished (figure 5) suggesting that other pathways participate in this response to high glucose. Nonetheless, the findings indicate that TSP1 expression is influenced by high glucose at the level of transcription and are consistent with observation reported by other investigators [38]. Overall, our observation that CTGF increases expression of ECM proteins, like collagens and TSP-1, support an important role for CTGF in ECM expression and bears important implications for tissue fibrosis as a consequence of hyperglycemia.

The role of CTGF in pathogenesis of a complex trait, like diabetic kidney disease, is unresolved. CTGF gene polymorphism has been linked to susceptibility to kidney disease in type 1 diabetes mellitus [61] and may also be a prognostic risk factor for cardiovascular outcomes in hemodialysis patients [62]. Yet, other report show no link between at least one polymorphism (-945G/C) in CTGF gene and kidney disease in types 1 and 2 diabetes [63], [64]. The findings of the current study further support a role for *ctgf* expression in the pathogenesis of diabetic kidney disease. In addition, we show that *ctgf* expression is an important mediator of ECM protein expression in response to high glucose. Future studies will be needed to further delineate the role of CTGF and signaling pathways in this response.

## Supporting Information

Figure S1 Food consumption (**A**), water intake (**B**), urine output (**C**) and body weight (**D**) in diabetic and non-diabetic wildtype (+/+) and heterozygous (+/−) mice after 4 months of STZ-induced diabetes mellitus. Values represent the mean ± standard deviation; *p<0.01; n = 8.
(TIF)

Figure S2 Masson-Trichrome stained sections of pancreas from C*tgf* wildtype (**A**) and heterozygous (**B**) mice. Immunohistochemical studies from wildtype (**C**) and heterozygous (**D**) identifying insulin-staining beta cells (yellow-red) and glucagon-staining alpha cells (green) in pancreatic islets. Exocrine pancreas (red) and DAPI stained nuclei (blue) are also shown in panels **C** and **D**; Magnification ×400.
(JPG)

Figure S3 Representative Masson-Trichrome-stained kidney sections from non-diabetic wildtype (+/+) [**A**] and CTGF heterozygous (+/−) [**B**] and diabetic wildtype [**C**] and CTGF heterozygous littermates [**D**] reveal tubular simplification with diabetes, without significant interstitial fibrosis.
(JPG)

Table S1 **Changes in expression of ECM-related genes in embryonic fibroblasts (MEF) derived from wild-type and knockout embryos following exposure to high glucose.** Expression was determined using an expression profiling kit (product #PM-012B, SA Biosciences). mRNA levels are expressed as mean ± SD relative to mRNA levels for glyceraldehye-3-phosphate dehydrogenase. NS – not significant.
(DOCX)
