# Supplementary figures and images for: Connective Tissue Growth Factor (CTGF) Expression Modulates Response to High Glucose
Source: PLoS One. 2013 Aug 12;8(8):e70441. doi: 10.1371/journal.pone.0070441 (PMC3741286; doi:10.1371/journal.pone.0070441)

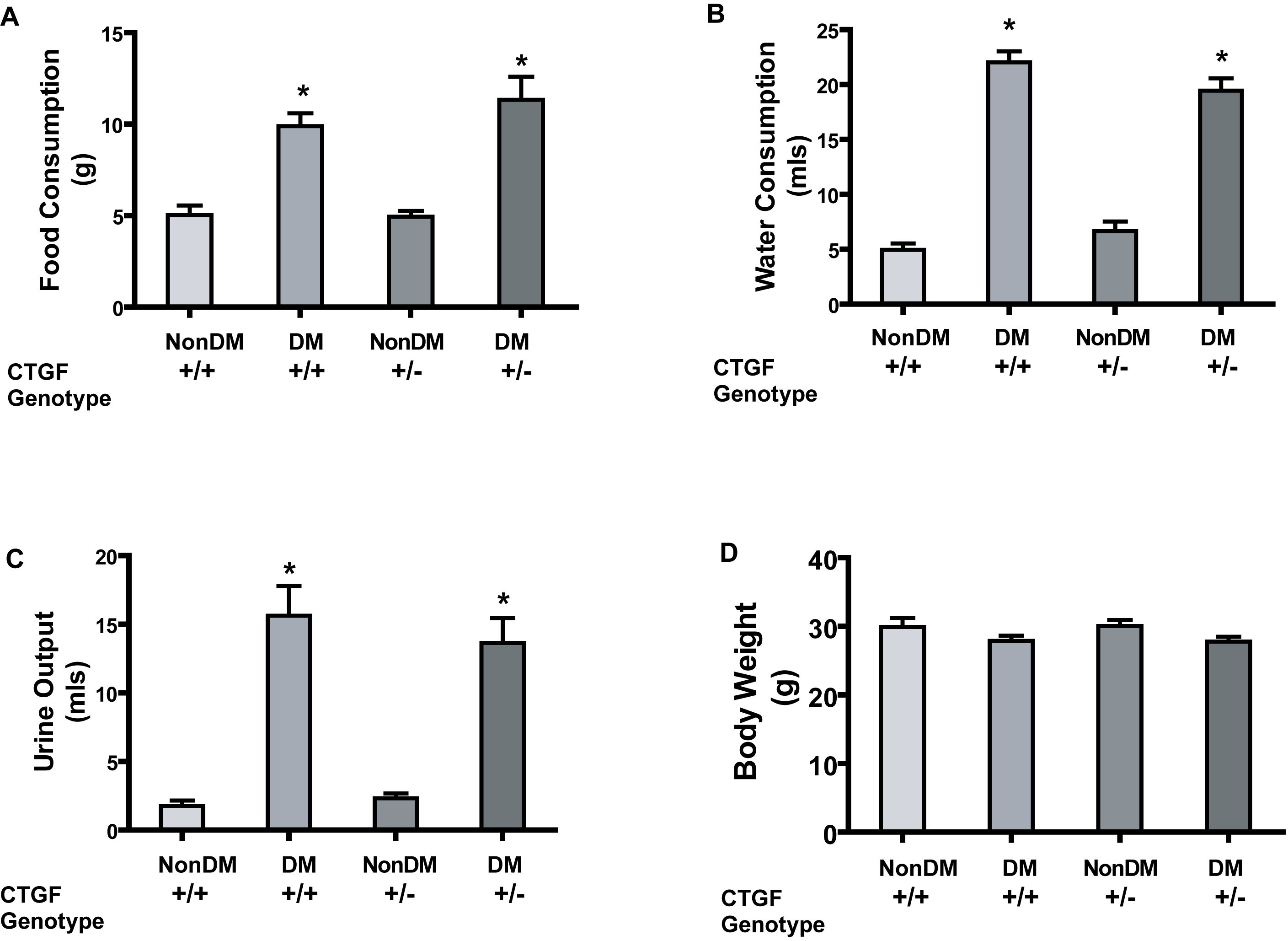

Supplement: Figure S1 — Food consumption (A), water intake (B), urine output (C) and body weight (D) in diabetic and non-diabetic wildtype (+/+) and heterozygous (+/−) mice after 4 months of STZ-induced diabetes mellitus. Values represent the mean ± standard deviation; *p<0.01; n = 8. (TIF) [file pone.0070441.s001.tif]

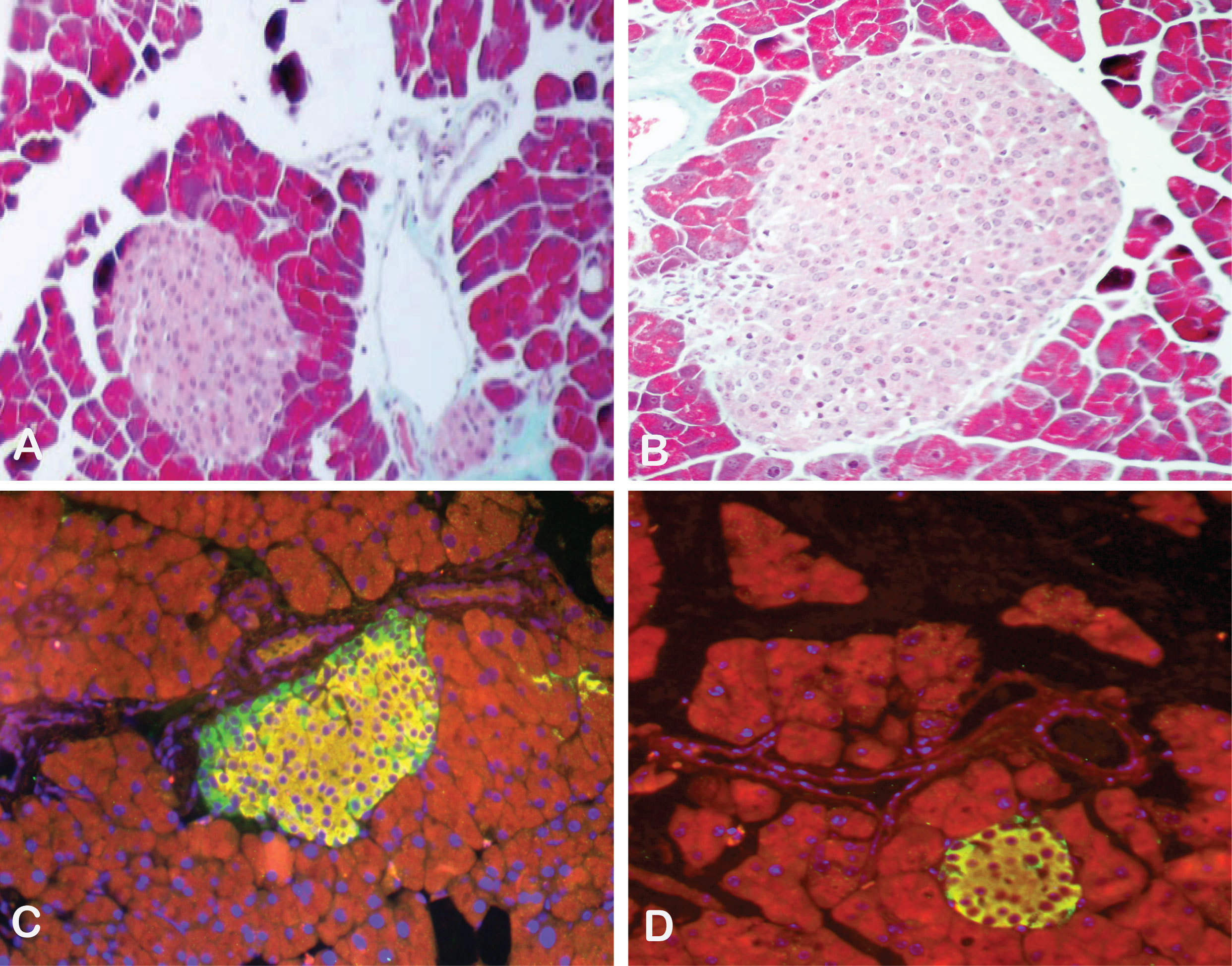

Supplement: Figure S2 — Masson-Trichrome stained sections of pancreas from Ctgf wildtype (A) and heterozygous (B) mice. Immunohistochemical studies from wildtype (C) and heterozygous (D) identifying insulin-staining beta cells (yellow-red) and glucagon-staining alpha cells (green) in pancreatic islets. Exocrine pancreas (red) and DAPI stained nuclei (blue) are also shown in panels C and D; Magnification ×400. (JPG) [file pone.0070441.s002.jpg]

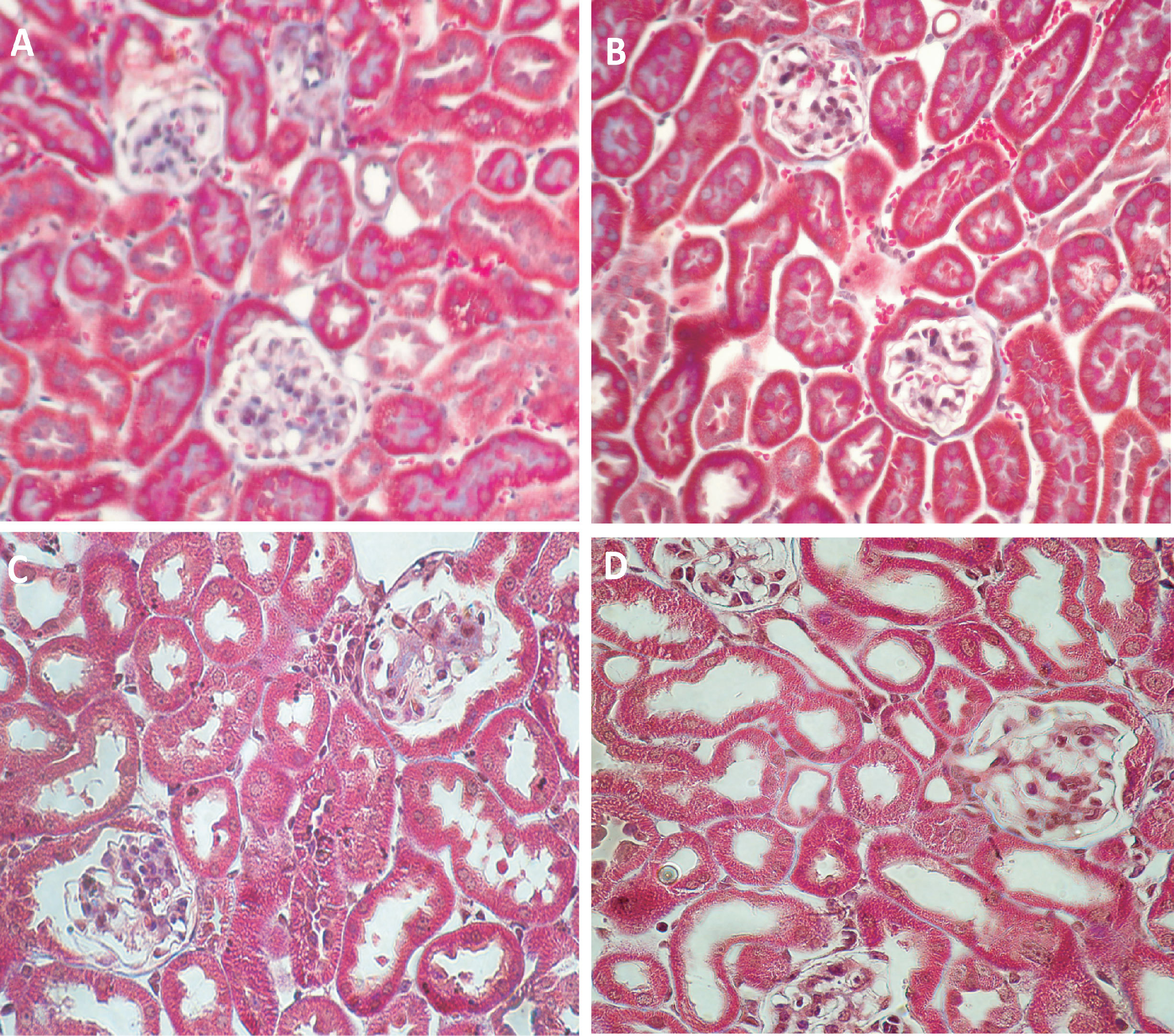

Supplement: Figure S3 — Representative Masson-Trichrome-stained kidney sections from non-diabetic wildtype (+/+) [A] and CTGF heterozygous (+/−) [B] and diabetic wildtype [C] and CTGF heterozygous littermates [D] reveal tubular simplification with diabetes, without significant interstitial fibrosis. (JPG) [file pone.0070441.s003.jpg]
