# Supplementary material for: Connective Tissue Growth Factor (CTGF) Expression Modulates Response to High Glucose
Source: PLoS One. 2013 Aug 12;8(8):e70441. doi: 10.1371/journal.pone.0070441 (PMC3741286; doi:10.1371/journal.pone.0070441)
Supplement: Table S1 — Changes in expression of ECM-related genes in embryonic fibroblasts (MEF) derived from wild-type and knockout embryos following exposure to high glucose. Expression was determined using an expression profiling kit (product #PM-012B, SA Biosciences). mRNA levels are expressed as mean ± SD relative to mRNA levels for glyceraldehye-3-phosphate dehydrogenase. NS – not significant. (DOCX) [file pone.0070441.s004.docx]

**Table S1**

|  |  | **Gene** | **Reference** | **Wildtype** | **Heteozygous** | **P** |
| --- | --- | --- | --- | --- | --- | --- |
|  |  | **Symbol** | **Sequence #** | **(+/+)** | **(+/-)** |  |
| Caveolin, caveolae protein | | Cav | NM_007616 | 1.08 ± 0.17 | 0.79 ± 0.19 | NS |
| Procollagen, type 1, alpha 1 | | Col1α1 | NM_007742 | 1.98 ± 0.03 | 1.12 ± 0.24 | P<0.05 |
| Procallegen, type IV, alpha 2 | | Col4 α2 | NM_009932 | 2.46 ± 0.22 | 1.39 ± 0.26 | P<0.02 |
| Procollagen, type XVIII, alpha 1 | | Col18 α1 | NM_009929 | 3.12 ± 0.51 | 1.65 ± 0.20 | P<0.02 |
| Extracellular matrix Protein 1 | | Ecm1 | NM_007899 | 0.96 ± 0.05 | 1.02 ± 0.02 | NS |
| Fibronectin 1 | | Fn1 | XM_129845 | 2.11 ± 0.42 | 1.22 ± 0.25 | P<0.05 |
| Laminin B1 subunit 1 | | Lamb1-1 | NM_008482 | 1.27 ± 0.21 | 0.98 ± 0.10 | NS |
| Laminin, gamma 1 | | Lamc1 | NM_010683 | 1.15 ± 0.22 | 0.97 ± 0.12 | NS |
| Secreted acidic cysteine rich | | Sparc | NM_009242 | 0.92 ± 0.14 | 1.21 ± 0.25 | NS |
| Glycoprotein | |  |  |  |  |  |
| Thrombospondin 1 | | TSP1 | NM_011580 | 3.81 ± 0.71 | 2.13 ± 0.21 | P<0.02 |
| Vitronectin | | Vtn | NM_011707 | 0.98 ± 0.03 | 0.95 ± 0.06 | NS |
